# Pharmacologic inhibition of LAT1 predominantly suppresses transport of large neutral amino acids and downregulates global translation in cancer cells

**DOI:** 10.1111/jcmm.17553

**Published:** 2022-09-07

**Authors:** Kou Nishikubo, Ryuichi Ohgaki, Hiroki Okanishi, Suguru Okuda, Minhui Xu, Hitoshi Endou, Yoshikatsu Kanai

**Affiliations:** ^1^ Department of Bio‐system Pharmacology, Graduate School of Medicine Osaka University Osaka Japan; ^2^ Integrated Frontier Research for Medical Science Division, Institute for Open and Transdisciplinary Research Initiatives (OTRI) Osaka University Osaka Japan; ^3^ Department of Applied Biological Chemistry, Graduate School of Agricultural and Life Sciences The University of Tokyo Tokyo Japan; ^4^ J‐Pharma Co., Ltd Yokohama Japan

**Keywords:** amino acid transporter, anti‐cancer agent, global translation, inhibitor, large neutral amino acids, LAT1, PDAC, SLC7A5

## Abstract

L‐type amino acid transporter 1 (LAT1; SLC7A5), which preferentially transports large neutral amino acids, is highly upregulated in various cancers. LAT1 supplies cancer cells with amino acids as substrates for enhanced biosynthetic and bioenergetic reactions and stimulates signalling networks involved in the regulation of survival, growth and proliferation. LAT1 inhibitors show anti‐cancer effects and a representative compound, JPH203, is under clinical evaluation. However, pharmacological impacts of LAT1 inhibition on the cellular amino acid transport and the translational activity in cancer cells that are conceptually pivotal for its anti‐proliferative effect have not been elucidated yet. Here, we demonstrated that JPH203 drastically inhibits the transport of all the large neutral amino acids in pancreatic ductal adenocarcinoma cells. The inhibitory effects of JPH203 were observed even in competition with high concentrations of amino acids in a cell culture medium. The analyses of the nutrient‐sensing mTORC1 and GAAC pathways and the protein synthesis activity revealed that JPH203 downregulates the global translation. This study demonstrates a predominant contribution of LAT1 to the transport of large neutral amino acids in cancer cells and the suppression of protein synthesis by JPH203 supposed to underly its broad anti‐proliferative effects across various types of cancer cells.

## 1 INTRODUCTION

Reprogrammed metabolism of nutrients, such as glucose, amino acids and lipids, is a general characteristic of cancers involved in their development and progression.^1^, ^2^ Amino acids and their metabolites are essential to fuel biosynthetic and bioenergetic reactions in cancer cells, including protein synthesis, nucleotide synthesis, lipogenesis and ATP production.^3^, ^4^ Furthermore, amino acids activate cellular signalling networks such as the mTORC1 (mechanistic target of rapamycin complex 1) pathway, which plays multiple pivotal roles in regulating cell survival, growth and proliferation and is often dysregulated in cancers.^5^, ^6^ Therefore, therapeutic intervention in amino acid utilization of cancer cells is a rational strategy for cancer treatment.^7^

The expression of several types of amino acid transporters is increased in cancer cells to fulfil the enhanced demands for amino acids.^8^ Among them, L‐type amino acid transporter 1 (LAT1; SLC7A5)^9^ is most highly and frequently upregulated in malignant tumours originating from various tissues/organs.^10^, ^11^ The transport process mediated by LAT1 is a Na^+^‐independent obligatory exchange of amino acids at 1:1 stoichiometry, in which large neutral amino acids (Met, Val, Leu, Ile, Phe, Tyr, Trp and His) are imported with high affinity^9^ being coupled to the efflux of cytoplasmic amino acids including non‐essential amino acids (NEAAs) with much lower affinity.^12^, ^13^ Therefore, LAT1 de facto contributes to the uptake of its extracellular substrates into cancer cells, including most of the essential amino acids (EAAs) that cannot be synthesized de novo. The high expression of LAT1 is associated with poor prognosis of patients and cancer cell proliferation in many cancer types, demonstrating the pathological importance of LAT1‐mediated amino acid transport in cancer.^10^, ^14^ In contrast, the expression of LAT1 in normal tissues/organs is considerably limited,^11^ for example in endothelial cells of the blood–brain barrier^15^ and syncytiotrophoblasts of the placenta.^16^

Accordingly, LAT1 has been recognized as a promising therapeutic and diagnostic target for cancers.^11^ Several LAT1 inhibitors have been developed as candidates for new anti‐cancer drugs that selectively restrict the supply of external amino acids into cancer cells.^17^, ^18^, ^19^, ^20^, ^21^, ^22^ Among the most evaluated and clinically advanced is a LAT1‐specific high‐affinity compound, JPH203 (KYT‐0353), obtained by the structural development from a thyroid hormone triiodothyronine (T3).^11^, ^22^ An accumulating number of pre‐clinical studies has reported the anti‐cancer effects of JPH203 both in vitro and in vivo. JPH203 inhibits the proliferation of various cancer cells of distinct tissues/organ origins.^22^, ^23^, ^24^, ^25^, ^26^, ^27^, ^28^, ^29^, ^30^, ^31^, ^32^ JPH203 also suppresses the migration^24^, ^25^, ^31^ and the cell cycle progression^23^, ^26^, ^28^ of cancer cells in vitro. Suppression of tumour growth by JPH203 in animal models has been demonstrated against colorectal cancer, thyroid cancer, biliary tract cancer, pancreatic cancer, breast cancer and T‐cell lymphoblastic lymphoma/T‐cell acute lymphoblastic leukaemia.^22^, ^23^, ^27^, ^28^, ^29^, ^33^, ^34^ In the first‐in‐human phase I clinical trial (UMIN000016546) conducted in patients with advanced solid tumours, JPH203 was well‐tolerated and provided promising activity against biliary tract cancer.^35^ A randomized phase II clinical trial of JPH203 against patients with advanced biliary tract cancers is currently ongoing in Japan (UMIN000034080).

Regardless of the progress towards its clinical application, fundamental pharmacological actions of JPH203 on cancer cells remain to be elucidated. For instance, the inhibitory effects of JPH203 (and other LAT1 inhibitors) on the amino acid transport in cancer cells have been evaluated only using a representative substrate Leu, mostly in Na^+^‐free buffered solutions previously.^22^, ^24^, ^25^, ^27^, ^28^, ^30^, ^31^, ^32^ However, cancer cells potentially express multiple Na^+^‐dependent and Na^+^‐independent amino acid transporters that exhibit a considerable overlap in the substrate selectivity.^8^ Therefore, it is still unclear whether the inhibition of LAT1 by JPH203 indeed achieves a broad suppression of the transport of large neutral amino acids, not only Leu, in cancer cells. The contribution of LAT1 to the overall transport of large neutral amino acids in cancer cells has not been clarified yet in former studies. Furthermore, it has not been demonstrated yet whether JPH203 exerts its inhibitory effects on the amino acid transport even in the presence of high concentrations of various amino acids, like in patients' body fluids, that would competitively interfere with and attenuate the inhibitory effects of JPH203 on LAT1.

The majority of carbon mass in macromolecules in rapidly proliferating cells is derived from amino acids.^36^ The primary anabolic process of amino acid metabolism is protein synthesis.^37^ As the rate of protein synthesis positively correlates with the proliferation rate of cells,^38^, ^39^ the downregulation of translation is likely one of the critical mechanisms of action underlying the anti‐proliferative effects of LAT1 inhibitors. Intriguingly, amino acids are not only utilized as building blocks of proteins but also stimulate signalling pathways, such as the mTORC1 pathway^5^, ^6^ and GAAC (general amino acid control) pathway,^40^, ^41^ that regulate global cap‐dependent translation initiation. Therefore, if LAT1 is primarily responsible for the uptake of large neutral amino acids in cancer cells, its pharmacologic inhibition potentially blocks the supply of amino acids both as biosynthetic materials and regulatory signals of protein synthesis. However, in previous studies, the influence of JPH203 on translation in cancer cells has been only surmised from its impact on amino acid signalling without assessing the protein synthesis itself (i.e. by referring to the altered phosphorylation levels in mTORC1‐ and GAAC‐pathways).^23^, ^24^, ^25^, ^27^, ^29^, ^31^

We conducted the present study to address the questions above, aiming to establish the pharmacological mechanisms of action of JPH203 as a novel anti‐cancer agent and further validate the clinical potential of LAT1 as a therapeutic target in cancer cells. Using pancreatic ductal adenocarcinoma (PDAC) cell lines as models, we revealed the drastic inhibition of the transport of all the preferred substrates of LAT1 by JPH203. We also verified the inhibitory effects of JPH203 in competition with high concentrations of LAT1‐substrate amino acids contained in the cell culture medium. Furthermore, analyses of protein synthesis based on the incorporation of puromycin into nascent polypeptides and the binding state of ribosomes with mRNA (polysome analysis) indicated the downregulation of global translation in cancer cells by targeting LAT1 with JPH203.

## 2 MATERIALS AND METHODS

### 2.1 Cell culture

Pancreatic cancer HPAC (CRL‐2119; ATCC), MIA PaCa‐2 (JCRB0070; JCRB), PANC‐1 (CRL‐1469; ATCC) and SUIT‐2 (JCRB1094; JCRB) cells were cultured in RPMI‐1640 supplemented with 10% FBS and 100 units/ml penicillin–100 μg/ml streptomycin. Cells were maintained in humidified incubator at 37°C supplied with 5% CO_2_.

### 2.2 Western blot

Cells were seeded at 3.0 × 10^5^ cells/dish in 100 mm dish containing 15 ml of medium and cultured for 48 h. Then, the cells were incubated for 24 h with a fresh medium containing 30 μmol/L JPH203 dihydrochlorides (JPH203, J‐Pharma Co., Ltd.) or the equivalent concentration of DMSO (0.3%, solvent for JPH203). Cells were washed with ice‐cold PBS, collected by centrifugation at 1500 × *g* for 5 min at 4°C, homogenized by sonication (UR‐21P; TOMY) and solubilized on ice for 30 min with Lysis buffer (50 mmol/L Tris–HCl [pH 7.4], 120 mmol/L NaCl, 20 mmol/L NaF, 1 mmol/L EDTA, 6 mmol/L EGTA, 20 mmol/L β‐glycerophosphate, 1 mmol/L PMSF, 1 mmol/L Na_3_VO_4_, Protease inhibitor cocktail tablets and 1% NP40). After centrifugation of the lysates at 21,000 × *g* for 15 min at 4°C, the supernatants were collected. Protein concentrations of the supernatants were determined using BCA Protein Assay Kit (Thermo Fisher Scientific). Proteins were resolved by SDS‐PAGE and transferred to PVDF Blotting Membrane (10600023; GE Healthcare Life science). The membranes were blocked at room temperature for 1 h with 5% skim milk/TBST and incubated overnight at 4°C with primary antibodies, followed by the incubation for 1 h at room temperature with horseradish peroxidase‐conjugated secondary antibodies. Chemiluminescent signals were detected by Amersham Imager 680 (GE Healthcare Life science) using Clarity Western ECL Substrate (BIO‐RAD).

Primary antibodies used are as follows: anti‐LAT1 (KE026, TransGenic); anti‐β‐actin (66009–1‐Ig) from Proteintech; anti‐phospho‐Thr389‐p70S6K (9234), anti‐phospho‐Ser235/236‐S6 ribosomal protein (4858), anti‐S6 ribosomal protein (2217), anti‐phospho‐Ser51‐eIF2α (3398), anti‐eIF2α (5324), anti‐Thr37/46‐phospho‐4E‐BP1 (2855) and anti‐4E‐BP1 (9452) from Cell Signalling Technology; anti‐p70S6K (sc‐230) from Santa Cruz Biotechnology.

### 2.3 Immunofluorescence staining

Cells were seeded at 5.0 × 10^4^ cells/well in 6 well plates containing collagen I‐coated coverslips and cultured for 48 h. Immunofluorescence staining and image acquisition were performed as described previously.^16^ The samples were observed with a confocal laser scanning microscope system LSM710 microscope (Zeiss). Antibodies used were as follows: anti‐LAT1 (KE026, TransGenic) and anti‐LAMP2 (H4B4, Developmental Studies Hybridoma Bank) as primary antibodies, and Alexa Fluor 488‐conjugated donkey anti‐rabbit IgG (A21206, Invitrogen) and Alexa Fluor 568‐conjugated donkey anti‐mouse IgG (A10037, Invitrogen) as secondary antibodies.

### 2.4 Cell proliferation assay

Cells were seeded at 1.0 × 10^3^ cells/well in 96‐well plates (100 μl of medium/well). The medium was replaced the next day with a fresh medium containing the indicated concentrations of JPH203. After 3 days of incubation, cell proliferation was measured by Cell Counting Kit‐8 (Dojindo). All the experiments were performed with eight well replicates per condition (*n* = 8).

### 2.5 Amino acid uptake measurement

Cells were seeded at 1.0 × 10^5^ cells/well on non‐coated 24 well plates and cultured for 48 h. After wash and preincubation for 5 min with Hanks' balanced salt solution (HBSS: 125 mmol/L NaCl, 4.8 mmol/L KCl, 1.2 mmol/L MgSO_4_, 1.2 mmol/L KH_2_PO_4_, 1.3 mmol/L CaCl_2_, 5.6 mmol/L d‐glucose and 25 mmol/L HEPES [pH 7.4]), cells were incubated for 1 min at 37°C in HBSS containing 1 μmol/L of radio‐labelled amino acid. For inhibition assays, 30 μmol/L JPH203 or the equivalent concentration of DMSO (0.3%) was added to HBSS. When indicated, Na^+^‐independent amino acid uptake was measured in Na^+^‐free HBSS, in which NaCl of HBSS was replaced with the same concentration of Choline‐Cl. Radio‐labelled amino acid (2 μmol/L) was added to RPMI‐1640 for the measurements in the cell culture medium. For all the conditions, uptake was stopped by washing with 3 ml ice‐cold HBSS. Then, the cells were lysed with 0.1 N NaOH and subjected to radioactivity measurements using a liquid scintillation cocktail ULTIMA GOLD XR (PerkinElmer) with a liquid scintillation counter (LSC‐5100; Aloka). Uptake values were normalized by the protein concentration of cell lysates determined by BCA Protein Assay Kit (Thermo Fisher Scientific). All the experiments were performed with four well replicates per condition (*n* = 4). Radiolabelled amino acids used are as follows: l‐[^14^C] Aspartic acid (Asp) [201 mCi/mmol, MC‐139], l‐[^14^C] Isoleucine (Ile) [325 mCi/mmol, MC‐174], l‐[^14^C] Leucine (Leu) [338 mCi/mmol, MC‐175] and l‐[^14^C] Phenylalanine (Phe) [448 mCi/mmol, MC‐262] from Moravek; l‐[14C] Glutamic acid (Glu) [220 mCi/mmol, ARC0165A], l‐[^14^C] Methionine (Met) [55 mCi/mmol, ARC0345] and l‐[^14^C] Tyrosine (Tyr) [482 mCi/mmol, ARC0655] from Muromachi Kikai; l‐[^14^C] Histidine (His) [109.7 mCi/mmol, NEC0857], l‐[^14^C] Valine (Val) [271 mCi/mmol, NEC291], l‐[^14^C] Tryptophan (Trp) [53.8 mCi/mmol, NEC367] from PerkinElmer.

### 2.6 Analysis of protein synthesis by puromycin labelling

Puromycin labelling of nascent polypeptides was performed basically as described previously.^42^ Cells were seeded at 3.0 × 10^5^ cells/dish in 100 mm dish containing 15 ml of medium and cultured for 48 h. Then, cells were incubated for 1.5 hr with 1 μmol/L puromycin in a fresh medium containing 30 μmol/L of JPH203 or the equivalent concentration of DMSO (0.3%). Puromycin incorporated into proteins was detected with an anti‐puromycin antibody (PEN‐MA001; Cosmo Bio) by Western blot.

### 2.7 Polysome analysis

Polysome analysis was performed basically as described previously.^43^ Cells were seeded at 8.0 × 10^5^ cells/dish in 150 mm dish containing 40 ml of medium and cultured for 48 h. The medium was replaced with a fresh medium containing 30 μmol/L JPH203 or the equivalent concentration of DMSO (0.3%). After 24 h of incubation, cells were treated with 100 μg/ml cycloheximide (CHX) for 5 min in medium, washed with ice‐cold PBS containing 100 μg/ml CHX and collected by centrifugation at 200 × *g* for 5 min at 4°C. The cells were suspended in hypotonic buffer (5 mmol/L Tris HCl [pH 7.4], 2.5 mmol/L MgCl_2_, 1.5 mmol/L KCl, 1 × protease inhibitor (EDTA‐free), 100 μg/ml cycloheximide, 2 mmol/L dithiothreitol, 200 unit/ml RNase inhibitor, 0.5% Triton X‐100 and 0.5% sodium deoxycholate). After centrifugation of the lysates at 4°C for 7 min at 16,000 × *g*, the supernatants were adjusted for RNA concentration at OD_260_. Sucrose gradient (5–45%) in 20 mmol/L HEPES [pH 7.6], 100 mmol/L KCl, 5 mmol/L MgCl_2_, 10 μg/ml CHX, 0.1 × protease inhibitor (EDTA‐free) and 8 units/ml RNase inhibitor was prepared in ultracentrifuge tubes (7042; SETON) using a gradient master (Biocomp). RNA samples (8.0 OD_260_ units, 500 μl) were loaded on the sucrose gradient and ultracentrifuged at 187,000 × *g* at 4°C for 2 h using L‐90K with SW32.1Ti rotor (Beckman Coulter). Polysome profiles were determined using Piston Gradient Fractionator (Biocomp) with continuous OD_254_ measurement (UV monitor AC‐5000; ATTO).

### 2.8 Data reproducibility and statistical analysis

All the experiments were repeated at least twice to ensure the reproducibility of the results. Statistical analyses were performed with GraphPad Prism9 (GraphPad software) by unpaired two‐tailed Student's *t*‐test. Differences were considered significant when *p*‐values were <0.05. *p < 0.05, **p < 0.01, ***p < 0.001, ****p < 0.0001, ns, not significant.

## 3 RESULTS

### 3.1 Expression of LAT1 in pancreatic cancer cells and the suppression of proliferation by JPH203

**FIGURE 1:**
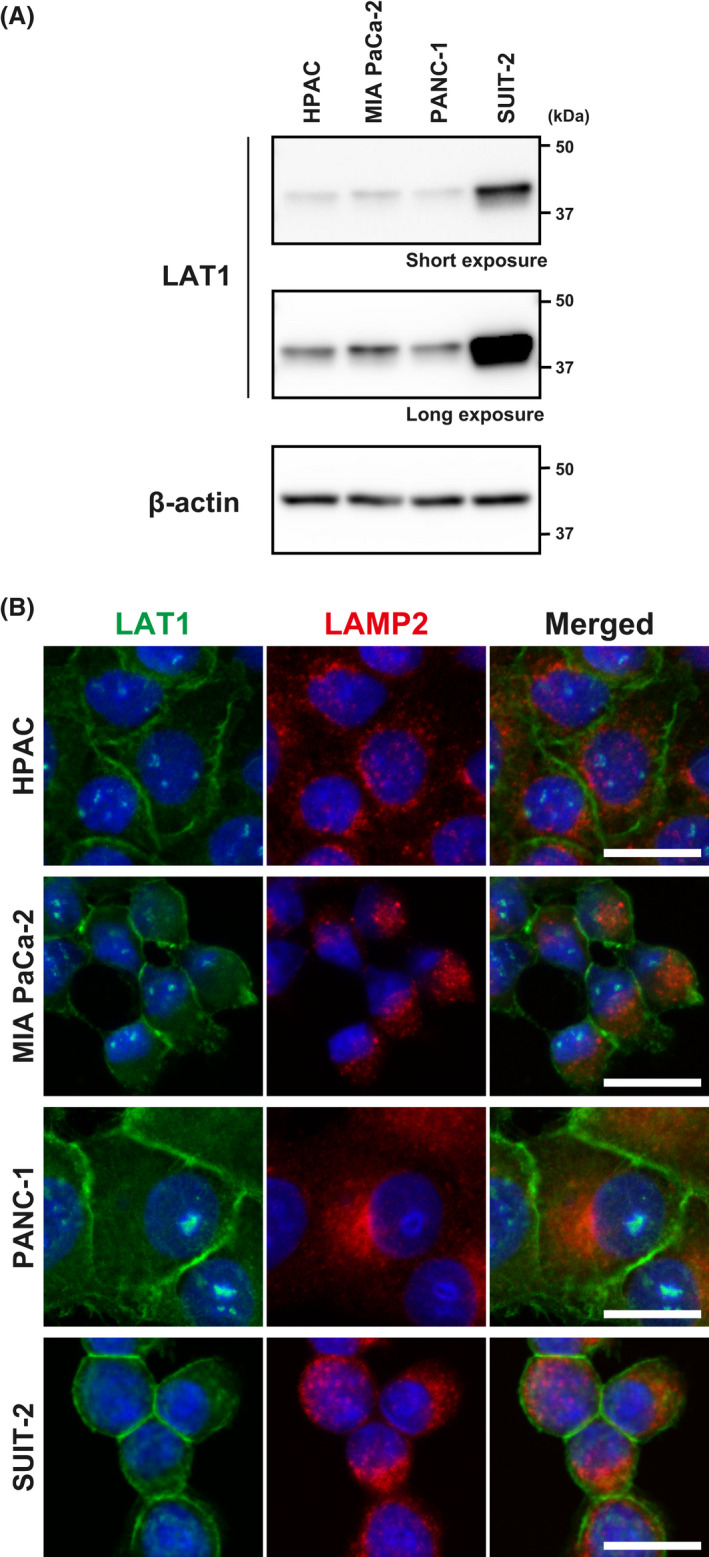
Expression and subcellular localization of LAT1 in pancreatic cancer cells. (A) LAT1 expression in HPAC, MIA PaCa‐2, PANC‐1 and SUIT‐2 cells analysed by Western blotting. β‐Actin; loading control. Short‐ and long‐exposure images are shown for LAT1. (B) Subcellular localization of LAT1 in HPAC, MIA PaCa‐2, PANC‐1 and SUIT‐2 cells analysed by immunofluorescence staining. LAMP2; lysosome marker. Nuclei stained with DAPI (blue) are shown in merge. Scale bar, 20 μm

**FIGURE 2:**
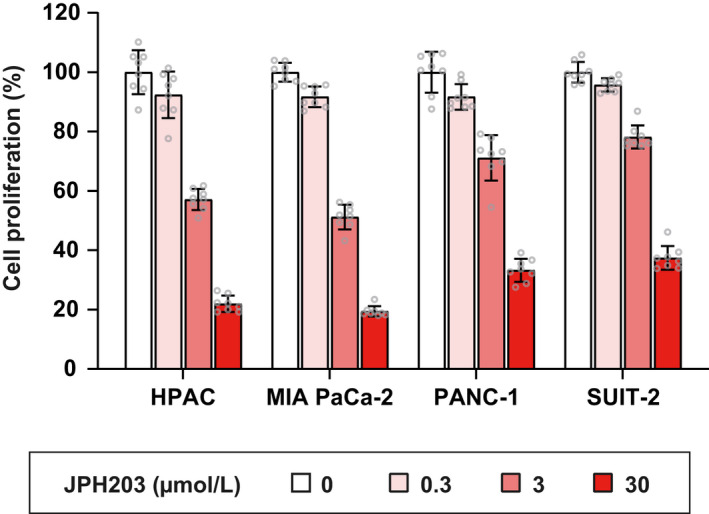
Inhibition of pancreatic cancer cell proliferation by JPH203. HPAC, MIA PaCa‐2, PANC‐1 and SUIT‐2 cells were cultured in the absence or the presence of JPH203 (0.3, 3 or 30 μmol/L) for 72 hr. Cell proliferation was measured by Cell Counting Kit‐8. Data were normalized for non‐treated controls (0 μmol/L) and shown as mean ± SD (*n* = 8)

LAT1 expression has been reported to positively correlates with cell proliferation index, angiogenesis and disease progression in pancreatic cancer.^44^, ^45^ However, the anti‐proliferative effects of JPH203 have not been established against PDAC cells. We thus selected PDAC cells in this study to investigate the functional importance of LAT1 for the amino acid uptake in cancer cells and the pharmacological effects of JPH203. All cell lines tested (HPAC, MIA PaCa‐2, PANC‐1 and SUIT‐2 cells) express LAT1, with the highest expression in SUIT‐2 cells (Figure 1A, Full blot in Figure S1), exhibiting a predominant localization on the plasma membrane (Figure 1B). No clear distribution of LAT1 was observed in lysosomes visualized with LAMP2, although LAT1 has been suggested to be partially present in lysosomes in other cell lines.^46^, ^47^ The four cell lines exhibited different proliferation rates in vitro, independent of the LAT1 expression levels (Figure S2). The addition of JPH203 to the cell culture medium inhibited the proliferation of all the PDAC cells in a concentration‐dependent manner (Figure 2). In all the following experiments, JPH203 was applied at 30 μmol/L, the highest dissolvable concentration of JPH203 in RPMI‐1640 medium.

### 3.2 Inhibition of the transport of all the large neutral amino acids by JPH203 in pancreatic cancer cells

**FIGURE 3:**
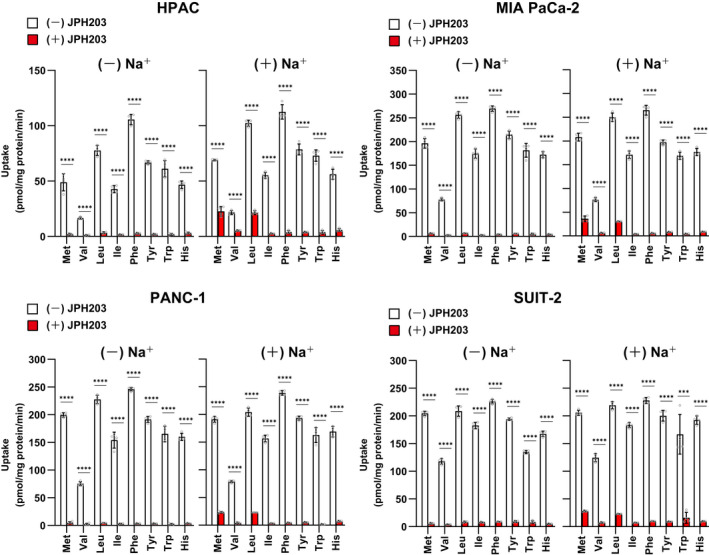
Effects of JPH203 on the uptake of large neutral amino acids in the absence or presence of Na^+^. Uptake of ^14^C‐labelled l‐amino acid (1 μmol/L) was measured in HPAC, MIA PaCa‐2, PANC‐1 and SUIT‐2 cells for 1 min in Na^+^ − free or Na^+^ − containing condition with or without JPH203 (30 μmol/L). Data are shown as mean ± SD (*n* = 4)

We then investigated the inhibitory effects of JPH203 on the transport of all the extracellular preferred LAT1‐substrates (i.e. Met, Val, Leu, Ile, Phe, Tyr, Trp and His) in PDAC cells. In the Na^+^‐free condition, in which the function of Na^+^‐dependent amino acid transporters is eliminated, treatment with JPH203 (30 μmol/L) almost entirely suppressed the uptake of all the eight amino acids (1 μmol/L) in the four cell lines (Figure 3, left bar graphs). Compared with the non‐treated control cells, the transport of each amino acid in the JPH203‐treated cells was decreased by 93%–97% in HPAC cells, 97%–98% in MIA PaCa‐2 cells, 97%–99% in PANC‐1 cells and 94%–97% in SUIT‐2 cells.

Even in the presence of Na^+^, JPH203 (30 μmol/L) profoundly suppressed the transport of Ile, Phe, Tyr, Trp, and His in the four cell lines (more than 90% reduction compared with the non‐treated controls). The transport of Val was also decreased by JPH203 by more than 90% in MIA PaCa‐2, PANC‐1 and SUIT‐2 cells, whereas the percentage of inhibition was slightly lower in HPAC cells (76%). Although the inhibition of Leu and Met transport by JPH203 was generally lower than that of the other six amino acids, it was reduced to 11% ~ 33% residual transport compared with the non‐treated controls (Figure 3, right bar graphs). It is especially of note that the presence of Na^+^ did not markedly increase the uptake of each amino acid in all the cell lines (Figure 3, white bars), indicating that the contribution of Na^+^‐dependent amino acid transporters is not predominant. These results demonstrate that the transport of all the large neutral amino acids in PDAC cells is primarily mediated by LAT1. In contrast, JPH203 did not influence the uptake of Asp and Glu, non‐substrate amino acids of LAT1, observed in HPAC and SUIT‐2 cells in the presence of Na^+^ (Figure S3). The acidic amino acid transport was not sufficiently high for quantitative analyses in MIA PaCa‐2 and PANC‐1 cells regardless of the presence of Na^+^ and in HPAC and SUIT‐2 cells in the absence of Na^+^.

### 3.3 Suppression of the amino acid transport by JPH203 in a cell culture medium

**FIGURE 4:**
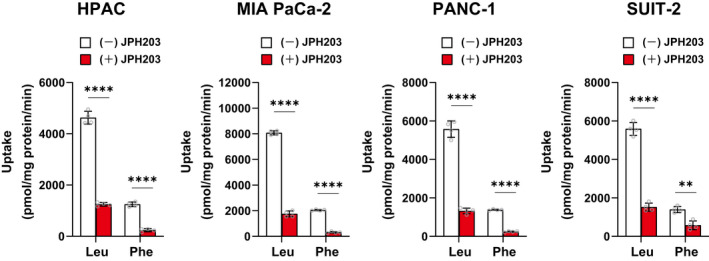
Effects of JPH203 on the uptake of l‐[^14^C] Leu and l‐[^14^C] Phe in cell culture medium. Uptake of l‐[^14^C] Leu (total 383 μmol/L; 381 μmol/L of cold in medium and 2 μmol/L of hot tracer) and l‐[^14^C] Phe (total 93 μmol/L; 91 μmol/L of cold in medium and 2 μmol/L of hot tracer) was measured in HPAC, MIA PaCa‐2, PANC‐1 and SUIT‐2 cells for 1 min in RPMI‐1640 medium with or without JPH203 (30 μmol/L). Data are shown as mean ± SD (*n* = 4)

Next, we evaluated the inhibitory effects of JPH203 on the amino acid uptake in PDAC cells in a cell culture medium, that is in the presence of high concentrations of various amino acids (Figure 4). RPMI‐1640 medium contains ~45 times higher concentration of large neutral amino acids in total (1356 μmol/L: Met 101 μmol/L, Val 171 μmol/L, Leu 381 μmol/L, Ile 381 μmol/L, Phe 91 μmol/L, Tyr 110 μmol/L, Trp 24 μmol/L and His 97 μmol/L) than that of JPH203 (30 μmol/L). Nevertheless, JPH203 prominently suppressed the transport of LAT1 substrates in the tested four cancer cell lines: the uptake of L‐[14C] Leu was reduced by 73, 78, 76 and 73%, and that of L‐[14C] Phe was reduced by 81%, 85%, 82% and 59% in HPAC, MIA PaCa‐2, PANC‐1 and SUIT‐2 cells, respectively.

### 3.4 Effect of JPH203 on amino acid signallings and protein synthesis

**FIGURE 5:**
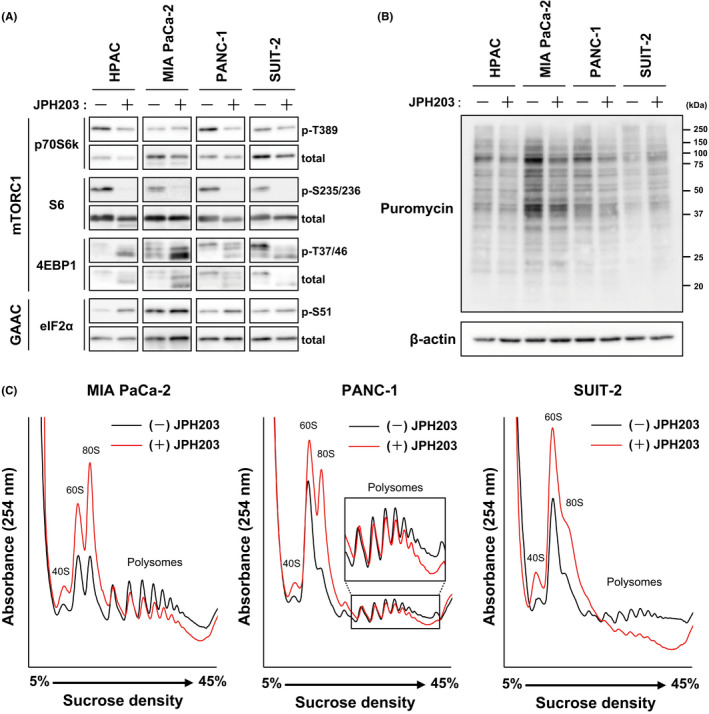
Effects of JPH203 on the amino acid signalling and cellular protein synthesis. (A) Phosphorylation of proteins involved in amino acid‐dependent regulation of translation was detected by Western blotting. HPAC, MIA PaCa‐2, PANC‐1 and SUIT‐2 cells were treated with JPH203 (30 μmol/L) for 24 hr and analysed for Western blotting. β‐Actin; loading control. (B) Protein synthesis was analysed by puromycin incorporation analysis in HPAC, MIA PaCa‐2, PANC‐1 and SUIT‐2 cells. After the treatment with JPH203 (30 μmol/L) and puromycin (1 μmol/L) for 1.5 hr, cell lysates were analysed by Western blotting using an anti‐puromycin antibody. β‐Actin; loading control. (C) Polysome analysis was performed using MIA PaCa‐2, PANC‐1, and SUIT‐2 cells treated with or without JPH203 (30 μmol/L) for 24 hr. Inset depicts a vertically expanded image of polysome fractions in PANC‐1 cells

Among the preferred substrates of LAT1, especially Leu is required to activate a serine/threonine kinase complex mTORC1 that positively regulates protein synthesis as one of the main downstream events.^5^, ^6^, ^48^ Even though the underlying mechanism was unknown, other LAT1‐substrates were also involved in the mTORC1 activation in a previous study.^49^ In addition, cellular amino acid availability reflected in the amounts of uncharged tRNAs controls the activity of the GAAC (general amino acid control) pathway that globally downregulates the cap‐dependent translation initiation under amino acid‐deficient conditions.^40^, ^41^ We thus examined the effects of JPH203 treatment on the amino acid signalling that regulate global protein synthesis by detecting phosphorylation levels of key factors in mTORC1‐ and GAAC‐pathways (Figure 5A). Treatment with JPH203 (30 μmol/L) generally decreased the phosphorylation of the downstream effectors of mTORC1 (p70S6K and S6 ribosomal protein), except p70S6K in MIA PaCa‐2 cells. 4EBP1 was detected as multiple bands, where the slower mobility represents the higher phosphorylation. The phosphorylation of 4EBP1 was also reduced by JPH203, as demonstrated by the migration of bands to lower molecular weights. In contrast, the phosphorylation of eIF2α in GAAC pathway was increased in all the four cell lines.

We then investigated the effect of LAT1 inhibition on the actual protein synthesis in cancer cells. First, we performed puromycin labelling of nascent polypeptides. An aminonucleoside antibiotic, puromycin, structurally resembles the 3′ end of aminoacyl‐tRNA. It enters the ribosomal A‐site and accepts an elongating polypeptide from peptidyl‐tRNA at P‐site. This reaction results in premature translation termination, leading to a release of peptidyl‐puromycin. Protein synthesis activity can be evaluated by adding low concentrations of puromycin into the cell culture medium and detecting the labelled polypeptides by Western blot using an anti‐puromycin antibody.^42^ As shown in Figure 5B, the inhibition of LAT1 by JPH203 (30 μmol/L) reduced the incorporation of puromycin into proteins in HPAC, MIA PaCa‐2 and PANC‐1 cells while increased in SUIT‐2 cells. The relative signal intensities of puromycin labelling in JPH203 treated cells compared with non‐treated control cells normalized by that of β‐actin were calculated to be 56.8%, 63.1%, 74.3% and 168.4% in HPAC, MIA PaCa‐2, PANC‐1 and SUIT‐2 cells, respectively.

We further evaluated the effects of JPH203 on protein synthesis by polysome analysis that fractionates mRNAs according to their ribosome binding status under sucrose density gradient centrifugation. The translation initiation and elongation rates determine the loading of ribosomes onto mRNA.^50^ Polysome‐associated mRNAs (mRNAs bound with multiple ribosomes) are actively translated, whereas monosome‐associated mRNAs (mRNAs bound with a single ribosome) are less translated. Treatment of MIA PaCa‐2, PANC‐1 and SUIT‐2 cells with JPH203 increased the peaks of 40S subunit, 60S subunit and monosome (80S), while oppositely reducing the peaks of polysome fractions (Figure 5C). The polysome fractions were not successfully detected in HPAC cells even in the control condition (data not shown).

Although the analyses of protein synthesis exhibited some inconsistent results or limitations depending on cell lines, the overall tendency in these results indicates that the inhibition of LAT1 attenuates the function of amino acids as regulatory signals in mTORC1 and GAAC pathways and downregulates the global translation in PDAC cells.

## 4 DISCUSSION

Our present study revealed the fundamental pharmacological actions of the representative LAT1 inhibitor JPH203. JPH203 significantly inhibited the transport of all the eight primary substrates of LAT1, not only Leu, in PDAC cells. The inhibitory effects were prominent and indifferent to the presence or absence of 125 mmol/L Na^+^ (mostly more than 90% inhibition, Figure 3). The relatively higher residual uptake for Met and Leu observed in JPH203‐treated cells in the presence of Na^+^ is likely reflecting the contribution of Na^+^‐dependent transporters. The uptakes of large neutral amino acids were not markedly increased by Na^+^. These results demonstrate the predominant contribution of LAT1 to the transport of all the large neutral amino acids in cancer cells. SLC6A14 (ATB^0,+^) that broadly accepts large neutral amino acids, as well as SLC38A2 (SNAT2), SLC38A3 (SNAT3) and SLC38A7 (SNAT7) that accept some large neutral amino acids such as His, Met and Leu are known to be upregulated in certain types of cancers.^8^ However, the contribution of such Na^+^‐dependent transporters, even if expressed, seems not prominent in the tested PDAC cells.

By using radio‐labelled l‐[^14^C] Leu and l‐[^14^C] Phe as tracers, we confirmed that JPH203 (30 μmol/L) suppresses the uptake of large neutral amino acids by mostly over 70%–80% even in the cell culture medium that contains 1356 μmol/L of large neutral amino acids in total (Figure 4). This is the first quantitative data set on inhibiting amino acid transport by JPH203 under the same experimental condition for cell proliferation assays. The result suggests the great potential of intravenously administrated JPH203 to sufficiently inhibit LAT1 in vivo even in competition with endogenous amino acids existing in tumour interstitium. To further assess this possibility, it will be necessary to obtain the time‐course of JPH203 concentration in tumour interstitium after JPH203 administration and estimate the expected inhibition of the transport on cancer cells.

Lastly, we asked whether the downregulation of translation is underlying the anti‐proliferative effects of LAT1 inhibitors or not. As shown in Figure 5A, JPH203 treatment affects amino acid signalling mediated by the mTORC1‐ and GAAC‐pathways that control translation. The phosphorylation of p70S6K (Thr389), S6 ribosomal protein (Ser235/236) and 4EBP1 (Thr37/46) was generally decreased by JPH203 in all the tested cell lines, indicating the reduced kinase activity of mTORC1. p70S6K phosphorylation in MIA PaCa‐2 cell did not decrease by JPH203, whereas the phosphorylation of 4EBP1, the other substrate of mTORC1, and that of S6 ribosomal protein, a downstream effector of p70S6K, decreased upon JPH203 treatment. The reason for this apparent discrepancy is not clear. One possibility could be compensatory phosphorylation of p70S6K (Thr389) by other kinases, which cannot fully activate the kinase activity of p70S6K due to its complex regulatory mechanism by cumulative phosphorylation at multiple sites.^51^, ^52^, ^53^, ^54^, ^55^ The increased phosphorylation of eIF2α by JPH203 in all the four cell lines indicates that the GAAC pathway was activated, suggesting that uncharged‐tRNA was increased in response to the amino acid deficiency by LAT1 inhibition. The overall alterations in the phosphorylation of mTORC1‐ and GAAC1‐pathways indicate that JPH203 suppresses cap‐dependent translation initiation through the amino acid signalling pathways in cancer cells.

Although a similar influence of JPH203 on amino acid signalling has already been shown in previous studies, the effects on actual translation have not been evaluated yet.^23^, ^24^, ^25^, ^27^, ^29^, ^31^ A most widely adopted method to investigate the translational activity is metabolic labelling with radio‐labelled amino acids such as l‐[^14^C] Leu and l‐[^35^S] Met/Cys. However, this approach is not adequate for our purpose because both Leu and Met are the preferred substrates of LAT1. LAT1 inhibition would overestimate the alteration in actual translational activity due to the reduced intracellular pool of tracers caused by LAT1 inhibition. Therefore, we used the two distinct methods in this study. In the puromycin labelling experiments, LAT1 inhibition by JPH203 reduced the amount of puromycin incorporation into nascent polypeptides in HPAC, MIA PaCa‐2 and PANC‐1 cells. In polysome analysis, JPH203 treatment decreased the translationally active polysome fractions, while increasing 40S subunit, 60S subunit and monosome fractions in MIA PaCa‐2, PANC‐1 and SUIT‐2 cells. These results indicate that LAT1 inhibition by JPH203 prevents the global translation initiation through the amino acid signalling mediated by mTORC1‐ and GAAC‐pathways and indeed suppresses the protein synthesis activity in PDAC cells.

It is of note that the analyses of protein synthesis gave inconsistent results or had limitations depending on cell lines. The puromycin labelling of protein was not decreased but increased by JPH203 treatment in SUIT‐2 cells, despite the apparent change in the phosphorylation in mTORC1‐ and GAAC‐pathways and polysome analysis (Figure 5A,C). The reason for this discrepancy among the analytical methods is unclear. A previous study alerted that puromycin labelling is not applicable for measuring protein synthesis in energetically challenged cells.^56^ Branched‐chain amino acids can replenish the TCA cycle through their metabolites (acetyl‐CoA and succinyl‐CoA). Inhibition of LAT1 may affect the energetic state of SUIT‐2 cells and compromise the assay by puromycin labelling. The considerably high expression of LAT1 (Figure 1A) and/or the particularly prominent effect of JPH203 on the binding state of ribosomes and mRNA in SUIT‐2 cells (Figure 5C) may have implications on the mechanism behind the increased incorporation of puromycin by JPH203 treatment. However, the details remain to be elucidated in further studies. Also, the polysome fractions were not detectable in HPAC cells for unknown reasons. The results of our study warn that the combinational application of the multiple methods is fundamentally important to characterize not only the effects of LAT1 inhibitors but also that of other amino‐acid targeted manoeuvres on the cellular protein synthesis activity.

The high LAT1 expression correlates with disease progression and poor prognosis of pancreatic cancer patients.^44^, ^45^ Pancreatic cancer is one of the most aggressive and lethal malignancies, with an overall 5‐year survival rate of ~10%.^57^ Although surgery is the only curative treatment, most patients are diagnosed with unresectable locally advanced or metastatic diseases. Monotherapy and combination therapy using gemcitabine have been the first‐line treatment for such patients. However, the overall survival can be only moderately prolonged.^58^, ^59^ Many patients develop drug resistance and experience severe adverse effects.^60^ Therefore, there is a desperate need to develop more effective therapeutic options.^61^ We recently demonstrated that the intravenously administered JPH203 significantly suppressed the growth of xenografted tumours of PDAC‐derived MIA PaCa‐2 cells.^33^ In addition to cancer cells, we also revealed a pathological upregulation of LAT1 in tumour‐associated endothelial cells. We showed that the anti‐angiogenic effects through the inhibition of endothelial LAT1 contributed to the in vivo anti‐cancer effects of JPH203. Therefore, it has been required to examine whether the inhibition of LAT1 in PDAC cells suppresses their proliferation by directly limiting cellular amino acid uptake. The present study showed that LAT1 is expressed in PDAC cell lines (Figure 1), and JPH203 successfully inhibits their proliferation in concentration‐dependent manners (Figure 2). Although more cell lines should be analysed to draw a general conclusion, there seemed to be no clear correlation between the expression level of LAT1 with the cell proliferation rate (Figure S2) or with the sensitivity to JPH203 (Figure 2). The extent to which amino acids transported by LAT1 contribute to cell proliferation may differ between cell lines. Sensitivity to LAT1 inhibitors is also considered not to be defined simply by the expression level of LAT1. In any case, the present study provides evidence to support the potential of LAT1 as a promising target for the treatment of PDAC. In this regard, we would like to emphasize that the expected cancer‐specific cytostatic anti‐proliferative effects of LAT1 inhibitors,^11^, ^26^ when used with gemcitabine, may pave the way to mitigate the adverse effects and resistance caused by genotoxicity and cytotoxicity of gemcitabine.

In summary, this study presents substantial evidence of the importance of LAT1 in the cellular uptake of large neutral amino acids in cancer cells. It contributes to developing a novel class of anti‐cancer agents targeting amino acid transporters in cancer cells. The inhibition of large neutral amino acid uptake and downregulation of global protein synthesis are the fundamental pharmacologic effects underlying the anti‐proliferative action of the LAT1 inhibitor JPH203. Although these findings need to be assessed for the general significance in other cancer cells from other tissue origins in the future, our present study provides basal pharmacological information to understand the mechanisms of action of JPH203 and other LAT1 inhibitors.

## AUTHOR CONTRIBUTIONS

**Kou Nishikubo:** Conceptualization (equal); data curation (lead); formal analysis (lead); investigation (lead); methodology (lead); validation (lead); visualization (equal); writing – original draft (supporting). **Ryuichi Ohgaki:** Conceptualization (equal); funding acquisition (supporting); project administration (equal); resources (supporting); supervision (lead); visualization (equal); writing – original draft (lead); writing – review and editing (equal). **Hiroki Okanishi:** Funding acquisition (supporting); resources (supporting); writing – review and editing (supporting). **Suguru Okuda:** Funding acquisition (supporting); resources (supporting); writing – review and editing (supporting). **Minhui Xu:** Funding acquisition (supporting); writing – review and editing (supporting). **Hitoshi Endou:** Resources (supporting); writing – review and editing (supporting). **Yoshikatsu Kanai:** Conceptualization (supporting); funding acquisition (lead); project administration (equal); resources (lead); supervision (supporting); writing – review and editing (equal).

## FUNDING INFORMATION

This research was financially supported by JSPS Grants‐in‐Aid for Scientific Research to Y.K. [19H03407], the Project for Cancer Research and Therapeutic Evolution from AMED (to Y.K. [JP20cm0106151] and [JP21cm0106151]), the Advanced Research for Medical Products Mining Program of NIBIO (to Y.K. [12–02]), the Translational Research Network Program from Ministry of Education, Culture, Sports, Science and Technology (to Y.K. [H25B7]) and Collaborative Research Grant from J‐Pharma Co., Ltd.

## CONFLICT OF INTEREST

Y.K. received a collaborative research grant from J‐Pharma Co., Ltd. H.E. founded J‐Pharma Co., Ltd. and has led the development of JPH203. Other authors declare no competing interests.

## Supporting information


Appendix S1

## Data Availability

All the data analyzed and presented in this study are available from the authors upon reasonable request.
