# Supplementary material for: Pharmacologic inhibition of LAT1 predominantly suppresses transport of large neutral amino acids and downregulates global translation in cancer cells
Source: J Cell Mol Med. 2022 Sep 7;26(20):5246–56. doi: 10.1111/jcmm.17553 (PMC9575050; doi:10.1111/jcmm.17553)
Supplement: Supplementary file 1 — Appendix S1 [file JCMM-26-5246-s001.pdf]

Supplementary figures

Pharmacologic inhibition of LAT1 predominantly suppresses transport of large neutral amino acids and downregulates global translation in cancer cells

Kou Nishikubo[1], Ryuichi Ohgaki[1, 2], Hiroki Okanishi[1], Suguru Okuda[1, 3], Minhui Xu[1], Hitoshi Endou[4], Yoshikatsu Kanai[1,2]

[1]Department of Bio-system Pharmacology, Graduate School of Medicine, Osaka University, Osaka, Japan

[2]Integrated Frontier Research for Medical Science Division, Institute for Open and Transdisciplinary Research Initiatives (OTRI), Osaka University, Osaka, Japan

[3]Department of Applied Biological Chemistry, Graduate School of Agricultural and Life Sciences, The University of Tokyo, Tokyo, Japan

[4]J-Pharma Co., Ltd., Yokohama, Japan

Correspondence

Yoshikatsu Kanai and Ryuichi Ohgaki, 2-2 Yamadaoka, Suita, Osaka 565-0871, Japan.

Email: ykanai@pharma1.med.osaka-u.ac.jp, ohgaki@pharma1.med.osaka-u.ac.jp

TEL: 06-6879-3521

FAX: 06-6879-3529

Supplementary figure S1

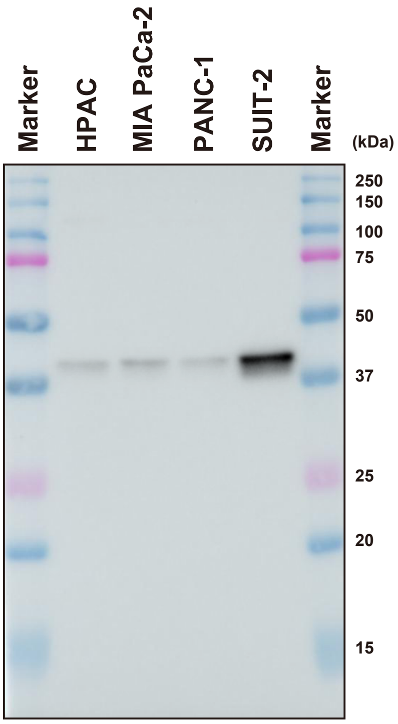

Figure S1

LAT1 expression in pancreatic cancer cells. Full blot image of Fig. 1. LAT1 expression in HPAC, MIA PaCa-2, PANC-1, and SUIT-2 cells detected by western blotting with short exposure. Positions of pre-stained molecular weight markers are also shown in merge.

Supplementary figure S2

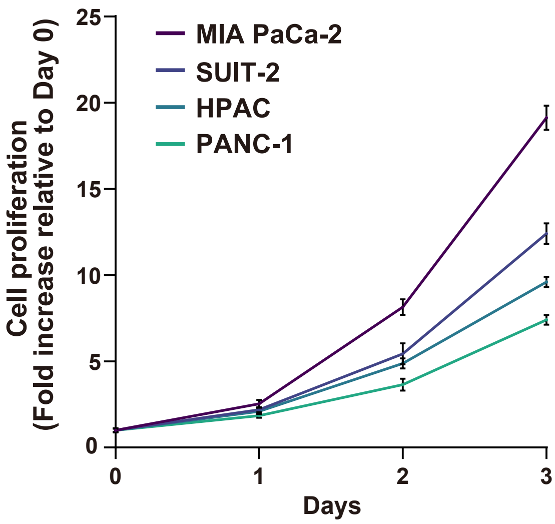

Figure S2

Proliferation of pancreatic cancer cells. HPAC, MIA PaCa-2, PANC-1, and SUIT-2 cells were cultured for 3 days (72 hr). Cell proliferation was measured by Cell Counting Kit-8 every day to calculate the fold increase relative to Day 0. Data were shown as mean ± SD (*n* = 8).

Supplementary figure S3

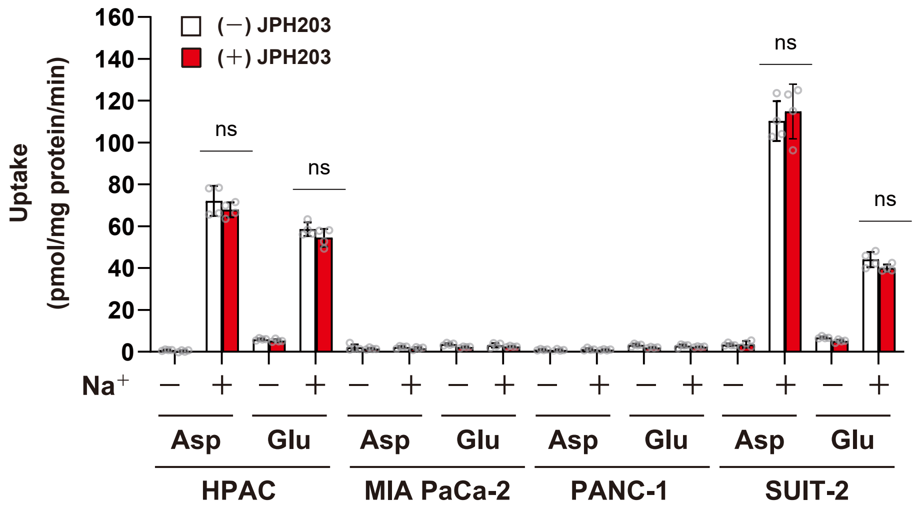

Figure. S3

Effects of JPH203 on the uptake of acidic amino acids in the absence and presence of Na^+.

Uptake of L-[^{14}C] Asp and L-[^{14}C] Glu (1 μmol/L) was measured in HPAC, MIA PaCa-2, PANC-1, and SUIT-2 cells for 1 min in Na^+-free (-) or Na^+-containing (+) HBSS with or without JPH203 (30 μmol/L). Data are shown as mean ± SD (*n* = 4).
